# Body Composition QTLs Identified in Intercross Populations Are Reproducible in Consomic Mouse Strains

**DOI:** 10.1371/journal.pone.0141494

**Published:** 2015-11-09

**Authors:** Cailu Lin, Brad D. Fesi, Michael Marquis, Natalia P. Bosak, Maria L. Theodorides, Mauricio Avigdor, Amanda H. McDaniel, Fujiko F. Duke, Anna Lysenko, Amin Khoshnevisan, Brian R. Gantick, Charles J. Arayata, Theodore M. Nelson, Alexander A. Bachmanov, Danielle R. Reed

**Affiliations:** Monell Chemical Senses Center, Philadelphia, PA, 19104, United States of America; National Institute of Genetics, JAPAN

## Abstract

Genetic variation contributes to individual differences in obesity, but defining the exact relationships between naturally occurring genotypes and their effects on fatness remains elusive. As a step toward positional cloning of previously identified body composition quantitative trait loci (QTLs) from F_2_ crosses of mice from the C57BL/6ByJ and 129P3/J inbred strains, we sought to recapture them on a homogenous genetic background of consomic (chromosome substitution) strains. Male and female mice from reciprocal consomic strains originating from the C57BL/6ByJ and 129P3/J strains were bred and measured for body weight, length, and adiposity. Chromosomes 2, 7, and 9 were selected for substitution because previous F_2_ intercross studies revealed body composition QTLs on these chromosomes. We considered a QTL confirmed if one or both sexes of one or both reciprocal consomic strains differed significantly from the host strain in the expected direction after correction for multiple testing. Using these criteria, we confirmed two of two QTLs for body weight (*Bwq5-6*), three of three QTLs for body length (*Bdln3-5*), and three of three QTLs for adiposity (*Adip20*, *Adip26* and *Adip27*). Overall, this study shows that despite the biological complexity of body size and composition, most QTLs for these traits are preserved when transferred to consomic strains; in addition, studying reciprocal consomic strains of both sexes is useful in assessing the robustness of a particular QTL.

## Introduction

The rationale for this project was born from challenges in mapping obesity genes. Spontaneously occurring mutations in a single gene are sufficient to radically alter body size, body length, and obesity [1, 2]. However, these are rare in humans and therefore not relevant to milder and more common forms of obesity. Another method to investigate the genetic substrates of obesity is through the systematic study of knockout mouse strains. Surveys of these genetically engineered mice indicate that up to one-third of viable knockout mouse strains differ in body weight or composition from their wild-type littermates [3, 4]. These studies indicate that targeted null mutations of genes can often affect body composition, but they are an imperfect guide to understanding how naturally occurring variation might do so. In fact, data from genetically engineered mice can lead investigators to misattribute genotype-phenotype relationships especially for obesity. For instance, human genome wide association results initially implicated the FTO gene, and the reduced body size of FTO knockout mice was thought to support this causal attribution [5]. However, subsequent studies suggested that variation in a different gene located nearby probably accounts for the original obesity effect, and that FTO is one of many genes that lead to reduce body size when inactivated [6].

A way to study naturally occurring variation is by using inbred mouse strains, which differ in adiposity indices, like gonadal fat depot weight, up to 20-fold [7, 8]. These strains can be intercrossed and the genome surveyed for regions shared more often than expected among larger or fatter mice. These studies have given rise to the identification of hundreds of influential genomic regions, quantitative trait loci (QTLs), as catalogued in the Mouse Genome Database [9], but few if any genes or their variants within these broad regions have been proven unequivocally to be responsible for the original association. A common prerequisite for a successful effort to identify gene variants underlying QTLs is that they survive the transfer to a fixed genetic background so that the informative region can be reduced until the gene variant itself is identified, a process known as positional cloning using congenic strains. This breeding method was pioneered in the 1940s and led to the identification of immunology genes [10]. A drawback to this method is that QTLs originally defined on a mixed genetic background of crosses between inbred mouse strains may not have the same effect when moved to a fixed genetic background of congenic strains. Therefore, it is useful to check in advance whether the QTL survives transfer to a fixed genetic background before the lengthy process of congenic mapping is undertaken.

One way to perform this check is to create chromosome substitution (consomic) strains, whereby one chromosome from a progenitor of the original intercross is transferred by breeding into the genome the other progenitor strain (i.e., the donor chromosome is integrated into the host strain) [11, 12]. The rationale for this approach is that if the QTL is confirmed in a consomic strain, congenic strains can then be efficiently created because most of the needed backcrossing has been completed during production of the consomic strain. In addition, consomic strains serve as a safety net because if the QTL effect is lost during the creation of a congenic strain, additional congenic strains with different donor regions can be created directly from the consomic strain. Consomic mice have proven to be an important renewable mapping resource; however, because production of consomic mice is time-consuming, only a few sets of consomic mice have been generated, e.g., [13–17]. We recently have bred seven new consomic strains, which were used in this study.

The goal of our research program is to genetically dissect a form of mouse obesity that is mild, does not fully manifest until middle age, and is not dependent on a high-fat diet [18–22]. In previous studies, we intercrossed the larger and fatter C57BL/6ByJ (B6) strain with the leaner 129P3/J (129) strain and mapped many QTLs, eight of which we chose to focus on here: two for body weight (*Bwq5-6*) [19], three for body length (*Bdln3-5*)[19], and three for adiposity, *Adip20* (originally named *Adip5)*, *Adip26* and *Adip27* [20, 21]. Three of these QTLs were male-specific (*Bdln4-5 and Adip27*); none were female-specific.

These eight QTLs are contained on three chromosomes (Chr 2, 7 and 9) so we bred six reciprocal consomic strains for Chr 2, 7 or 9 derived from the B6 and 129 inbred strains. (By ‘reciprocal’ we mean that a B6 chromosome was transferred by serial backcrossing on the 129 genetic background and vice versa, two reciprocal strains in total for each donor chromosome). We also bred a consomic strain from Chr 1 to determine how often new QTLs can be detected in consomic strains that were *not* previously detected in intercross populations. Mouse husbandry and phenotyping procedures were similar in the original intercross studies and the current consomic study, e.g., mice were fed a low-fat chow diet and were housed in the same animal facility.

Thus, the specific objective of the current study was to determine if QTLs mapped in the intercross mice are replicated in consomic mice. We considered a QTL replicated if it has met the following criteria: (i) if there was significant difference from the inbred partner strain in at least one sex and at least one reciprocal strain, and (ii) if the direction of allelic effect was the same in the consomic strain as in the original intercross population. We did not require that the sex-specific pattern be discernable in the consomics for a QTL to be considered confirmed, but the consistency of sex effects are considered and discussed separately.

## Materials and Method

The Institutional Animal Care and Use Committee of the Monell Chemical Senses Center approved all procedures. All body composition data from this study are available through the Mouse Phenome Database at phenome.jax.org (dataset: Reed2) [23] and through dryad, http://dx.doi.org/10.5061/dryad.b3bn2.

### Inbred and consomic mouse strains

C57BL/6ByJ (B6) and 129P3/J (129) inbred mice were purchased from the Jackson Laboratory (Bar Harbor, ME) and used as founders of consomic mice and parents of inbred mice used for body composition analyses. Consomic strains were bred by serial backcrossing onto B6 or 129 host inbred strains to make the reciprocal strains. We also created and tested a consomic strain with a donor chromosome (Chr 1) for which no body composition QTL was previously reported in crosses between these specific B6 and 129 strains. In total we bred seven consomic strains: two reciprocal strains from each of three chromosomes (2, 7, and 9) and one non-reciprocal strain (Chr 1). The breeding strategy to produce these strains is summarized immediately below and reported in detail in a separate manuscript currently in preparation.

Female F_1_ hybrids between the B6 and 129 progenitor inbred strains were backcrossed to males from a host strain, B6 or 129 (which resulted in elimination of the Y chromosome from the donor strain) to generate the N_2_ backcross generation. In the N_2_ and subsequent backcross generations, male offspring with full-length (non-recombinant) target chromosomes derived from the donor strain were identified and backcrossed to females from the host inbred strain, B6 or 129. To facilitate elimination of the donor genome in non-target chromosomes using a "speed consomic" approach [24, 25] we conducted genome scans of the N_2_ and N_3_ backcross generations. Heterosomic males (those with one full-length donor chromosome) and the smallest proportion of residual donor genome were used as breeders of the next generation. From the N_4_ generation onward, markers in the donor chromosome were genotyped, and breeders were chosen if they retained the full-length donor chromosome (for the list of markers used to verify the full-length chromosome, see **S1 Table**). After reaching backcross generation N_6_—N_8_, heterosomic males and females were intercrossed, and their homosomic progeny (i.e., mice homozygous for the donor chromosome) was used to propagate the homozygous consomic strains. These strains were genotyped with a panel of markers to test for residual heterozygosity; the outcomes of these tests are in **S2 Table**. We had planned to phenotype 20 mice per strain and these target numbers were attained for all 6 strains included in the analyses, except in one case (129.B6-Chr2), which was not included in analyses.

### Mouse husbandry

All inbred and consomic mice used for body composition analyses were born and reared in the Monell Center Animal Facility. Pups were weaned at 21–30 days of age and reared in same-sex groups until they were 6 months of age, when they were euthanized and used for body composition analyses. All mice were housed in temperature-controlled rooms (23°C) with a 12:12 light cycle and had free access to water and pelleted Teklad Rodent Diet 8604.

### Body composition measures

Mice were euthanized by carbon dioxide affixation, weighed to the nearest 0.1 gram, measured for body length to the nearest tenth of a centimeter, and the gonadal fat depot was removed and weighed. Body length was initially measured with a ruler (65% of mice), which was later replaced with electronic calipers (Fowler ProMax, Kelley and Kelly Industrial Supply, Syracuse, NY).

### Statistical analysis

Each group of consomic mice was compared with the appropriate host strain and the expected significant differences between consomic and inbred partner strains were interpreted as the presence of a QTL. These comparisons were made by one-tailed t-test for body weight and body length, and by general linear models for adiposity (with strain as factor and body weight as covariate). We used a one-tailed t-test because the expected direction of effect was known in advance based on the original QTL. To match earlier methods [19–21], the adiposity analysis model included the weight of the adipose depot as the outcome measure and body weight as a covariate. To account for multiple tests, we applied a Bonferroni correction (three strains x three traits, N = 9, p = 0.0056). All data analyses were conducted with Statistica, version 12 (StatSoft, Tulsa, OK), and GraphPad Prism, version 6.01 for Windows (GraphPad Software, San Diego, CA).

## Results

### QTLs detected in original F_2_ studies

We begin by summarizing the previous QTL results from F_2_ mapping studies to facilitate interpretation of the new consomic results. Eight QTLs which are the focus of study here were previously identified in an F_2_ intercross between the B6 and 129 strains (**Table 1**). Five the QTLs (i.e., all of the body weight and body length QTLs) were named in the original manuscript and those are the names we use in this report, i.e., *Bwq5-6* and *Bdln3-5*. One QTL for adiposity was originally named *Adip5* but this name was later changed to *Adip20* (the name we use here) by the International Committee on Standardized Genetic Nomenclature for Mice. The two remaining QTLs were not named in the original reports but were retroactively named by this committee (*Adip26* and *Adip27)*.

**Table 1:** QTLs originally detected in F_2_ hybrids. F_2_%var = % trait variance in F_2_ explained by the QTL; estimates are not available (NA) for all QTLs from all prior studies. Ref = reference.

Type	Chr	QTL	Sex	B6 effect	QTL peak, cM^#^	Confidence interval, cM^#^	F_2_%var	Ref
Body weight	2	*Bwq5*	♂♀	increase	68	24 to 84	4.8	[19]
	9	*Bwq6*	♂♀	increase	84	68 to telomere	4.3	[19]
Body length	2	*Bdln3*	♂♀	increase	70	65 to 75	8.7	[19]
	9	*Bdln4*	♂	increase	56	30 to telomere	3.2	[19]
	9	*Bdln5*	♂	increase	70	30 to telomere	2.5	[19]
Adiposity*	2	*Adip26*	♂♀	decrease	75	48 to telomere	9.5	[19]
	7	*Adip27*	♂	increase	32	0 to 42	10.2	[21]
	7	*Adip27*	♂	increase	3	0 to 15	NA	[22]
	9	*Adip20* ^§^	♂♀	decrease	35	6 to 54	4.7	[19]
	9	*Adip20*	♂♀	decrease	46	17 to 49	NA	[20]
	9	*Adip20*	♂♀	decrease	18	0 to 72	NA	[21]
	9	*Adip20*	♂♀	decrease	28^^^	12 to 35	NA	[22]
	9	*Adip20*	♂♀	decrease	73^^^	60 to 80	MA	[22]

* Adiposity is defined as the weight of the gonadal adipose depot relative to body size [19–21]. However a study using percent body fat [22] is included here for reference because both these fatness measures are closely related and thus informative.

*# cM* positions as provided in the original report.

^*§*^ formerly referred to as *Adip5* [19, 20].

^ observed when mice were fed a high-fat diet (mice ate a low-fat mouse chow diet in the other studies referenced in this table).

These original QTLs differed in the direction and strength of their allelic effects. In six of eight QTLs, the B6 allele increased the trait, the exceptions being *Adip20* and *Adip26*. Some of the QTLs had overlapping confidence intervals which suggest they may arise from the same underlying variant, e.g., body length (*Bdln3*) and body weight (*Bwq5)* on chromosome 2. The amount of phenotypical variance in F_2_ accounted for by these QTLs ranged from 2.5 to 10.2%. Five of the QTLs were observed in both sexes, but three were not: *Bdln4*, *Bdln5* and *Adip27* were specific to males.

### Generation of consomic mice

We now turn to outcome of the breeding plan to produce the consomic mice. We tried to breed six reciprocal and one non-reciprocal strains for a total of seven strains. We were successful at breeding mice that had a full length donor chromosome and all mice tested in each strain had the expected donor-region genotype at all markers tested (see **S1 Table** for list of markers). We were also successful at breeding mice with low residual heterozygosity using the ‘speed method’. The residual heterozygosity for each strain ranged from 0.87% to 0.0% (**S2 Table)** and thus all strains have met the genetic purity requirements (<1% of residual amount of unlinked donor genome) for congenic and consomic strains [26, 27]. However not all aspects of the consomic breeding program were as successful. One strain (129.B6-Chr2) was very difficult to breed. Therefore there were not enough mice to be included in any statistical analysis of the body composition data. A list of the successful strains and the details including their full and abbreviated names, their availability in mouse repositories and associated catalog numbers, the number of generations of breeding, group sample sizes and ages are in **Table 2**. Our original goal was to breed 20 mice per strain and with the exception of the 129.B6-Chr2 strain, our breeding goals were met. By intention, mice were six months of age on average when measured for body composition, which previous work suggests is a time of maximal difference among the parent strains [18] and our standard practice when studying this obesity model.

**Table 2:** Inbred and consomic mouse strains used in this study. Abbreviations: F = female; M = male; M = mean; SD = standard deviation; d = days; n/a = not applicable

Official strain symbol	Abbreviation	MMRRC ID*	JAX ID*	Generations	N mice (F/M)	Age (M±SD; d)
129P3/J	129	n/a	000690	n/a	24/14	188±12
C57BL/6ByJ	B6	n/a	001139	n/a	21/21	184±5
129P3/J-Chr 1^C57BL/6ByJ^/MonMmjax^#^	129.B6-Chr1	036684	018675	N8F2-4, N9F4	1919	184±2
129P3/J-Chr 7^C57BL/6ByJ^/MonMmjax	129.B6-Chr7	036686	018677	N7F2-3	20/20	187±2
129P3/J-Chr 9^C57B6/ByJ^/MonMmjax	129.B6-Chr9	036687	018678	N7F3-5	10/11	183±4
C57BL/6ByJ-Chr 2^129P3/J^/MonMmjax	B6.129-Chr2	036688	018679	N7F2-4	16/15	197±9
C57BL/6ByJ-Chr 7^129P3/J^/MonMmjax	B6.129-Chr7	036689	018680	N6F7,9,13	20/20	192±14
C57BL/6ByJ-Chr 9^129P3/J^/MonMmjax	B6.129-Chr9	036690	018681	N6F9-10,13	20/19	200±15
Combined					150/139	190±11

* Identification numbers (ID) are shown for strains available from the Jackson Laboratory (JAX; http://jaxmice.jax.org) and the Mutant Mouse Regional Resource Center (MMRRC; https://www.mmrrc.org).

^#^ ‘Mon’ within mouse strain name is a laboratory code for the Monell Chemical Senses Center issued by the Institute for Laboratory Animal Research (ILAR; http://dels.nas.edu/ilar_n/ilarhome/labcode.shtml).

### Analysis of QTL replication in consomic mice

**Fig 1:**
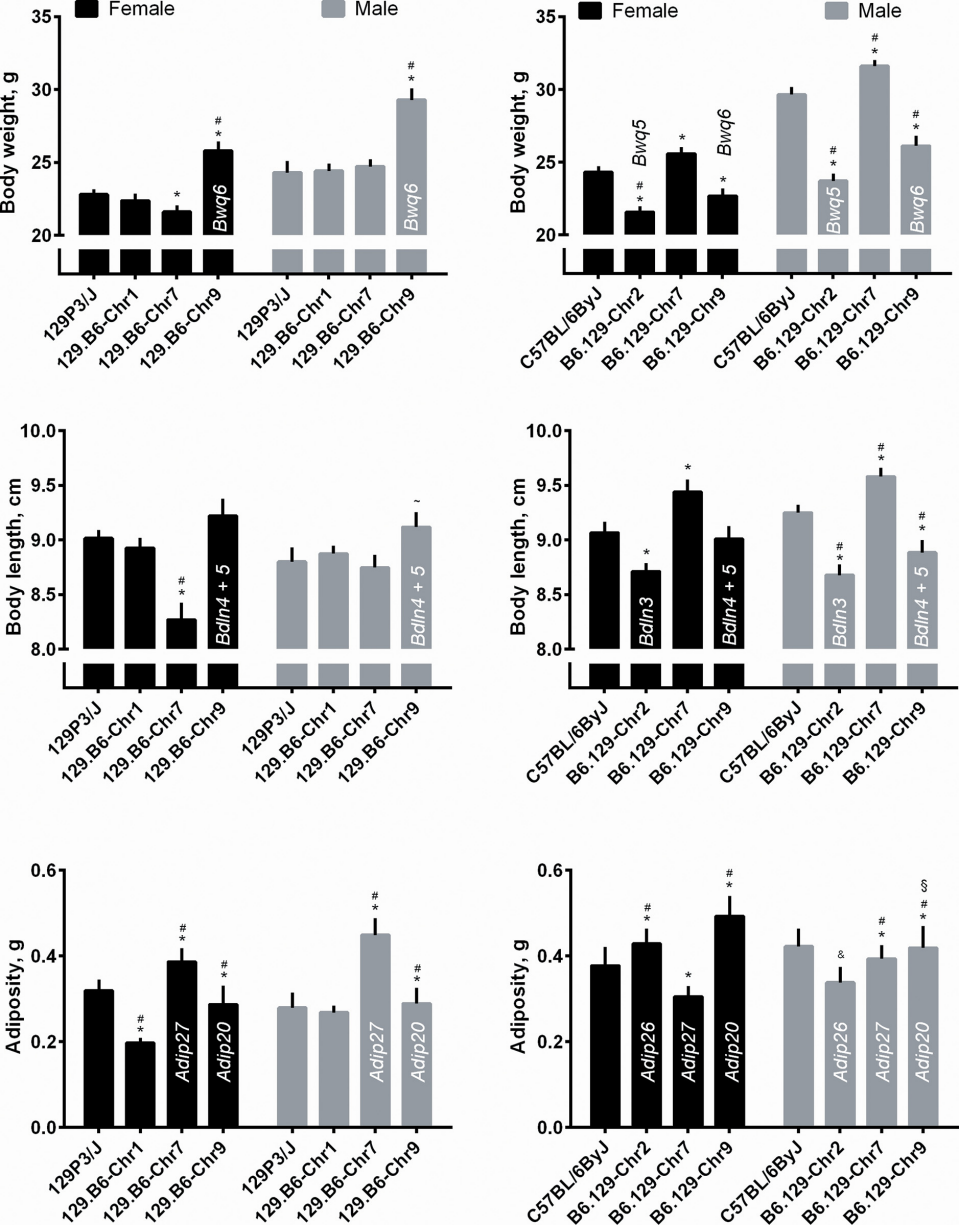
QTLs detection in consomic mice: Average values of body size and composition measures in inbred and consomics strains. Body weight (top), body length (middle), and adiposity (bottom) in inbred and consomic strains (means ± SEM). Left panels: Strains with 129 genetic background. Right panels: Strains with B6 genetic background. Asterisks (*) indicate a nominal difference between consomic strain and its inbred host (p < .0.05), # indicates significant after correction for multiple testing (p<0.0056). ^~^p = 0.0545. ^&^borderline significance. ^§^mice are heavier (top panel) but have similar gonadal weight, thus are leaner after adjustment for body weight.

The goal of this project was to determine whether QTLs are detectable when they are transferred to consomic strains and our method of detection is to compare the consomic to host strain for body weight, body length and adiposity. We expect these traits to be higher or lower than the host strain based on the direction of allelic effect of the original QTL. These differences in consomic-host strain means were evaluated by a t-test (for body weight and body length) and by a general linear model for adiposity. (The use of the general linear model for adiposity allowed us to assess fatness relative to body weight and was the method used to evaluate adiposity in prior studies of the F_2_ intercross). The results of the statistical tests by each QTL, sex and strain are shown in **Table 3** and **Fig 1**. Twenty five of the consomic-host strain comparisons are at least nominally significant (p<0.05; marked by an ‘*’ in **Fig 1**) and 19 comparisons are significant when the Bonferroni correction was applied (marked by a ^‘#^’).

**Table 3:** Body composition QTL affects in the consomic strains. F = female, M = male. Body weight and body length by t-test; adiposity. F-test (general linear model, strain as factor and body weight as covariate). Both statistical tests (t-test and general linear model) are one-tailed test because the expected direction of effect was known from original QTL studies of F_2_ hybrids.

Trait	129 strains	QTL	Sex	Test statistic	p-value	B6 strains	QTL	Sex	Test statistic	p-value
Body weight	129.B6-Chr1	*-*	F	t(41) = -0.7	0.2426	B6.129-Chr2	*Bwq5*	F	t(35) = -4.84	0.0000* ^#^
			M	t(31) = 0.12	0.451		* *	M	t(33) = -7.94	0.0000* ^#^
	129.B6-Chr7	*-*	F	t(41) = -2.12	0.0201*	B6.129-Chr7	*-*	F	t(39) = 2.11	0.0205*
		* *	M	t(32) = 0.46	0.3228		* *	M	t(38) = 2.85	0.0035* ^#^
	129.B6-Chr9	*Bwq6*	F	t(32) = 4.35	0.0001* ^#^	B6.129-Chr9	*Bwq6*	F	t(39) = -2.5	0.0083*
		* *	M	t(23) = 4.31	0.0001* ^#^		* *	M	t(37) = -4.1	0.0001* ^#^
Body length	129.B6-Chr1	*-*	F	t(40) = -0.78	0.2199	B6.129-Chr2	*Bdln3*	F	t(35) = -2.62	0.0064*
		* *	M	t(31) = 0.52	0.303		* *	M	t(32) = -4.83	0.0000* ^#^
	129.B6-Chr7	*-*	F	t(41) = -4.5	0.0000* ^#^	B6.129-Chr7	*-*	F	t(39) = 2.48	0.0088*
		* *	M	t(31) = -0.3	0.3836		* *	M	t(38) = 3.06	0.0020* ^#^
	129.B6-Chr9	*Bdln4*, *Bdln5*	F	t(31) = 1.36	0.0926	B6.129-Chr9	*Bdln4*, *Bdln5*	F	t(39) = -0.37	0.3574
		* *	M	t(23) = 1.67	0.0545^~^		* *	M	t(37) = -2.7	0.0051* ^#^
Adiposity	129.B6-Chr1	*-*	F	F(1, 40) = 17.52	0.0001* ^#^	B6.129-Chr2	*Adip26*	F	F(1, 33) = 7.68	0.0046* ^#^
		* *	M	F(1, 29) = 0.13	0.3597		* *	M	F(1, 32) = 7.58	0.9952^&^
	129.B6-Chr7	*Adip27*	F	F(1, 40) = 18.70	0.0000* ^#^	B6.129-Chr7	*Adip27*	F	F(1, 38) = 6.91	0.0061*
		* *	M	F(1, 31) = 19.39	0.0001* ^#^		* *	M	F(1, 37) = 8.21	0.0034* ^#^
	129.B6-Chr9	*Adip20*	F	F(1, 31) = 16.72	0.0001* ^#^	B6.129-Chr9	*Adip20*	F	F(1, 38) = 13.25	0.0004* ^#^
		* *	M	F(1, 22) = 10.00	0.0023* ^#^		* *	M	F(1, 36) = 8.18	0.0035* ^#^

* = significant, p < .05.

^#^ = significant after Bonferroni correction (3 strains x 3 phenotypes = 9 tests, p = 0.0056).

~, borderline significance p-value, p = 0.0545.

^&^ = one-tailed p-value arise from prediction (see **Table 1**) that this consomic strain (B6.129-Chr2) would have higher adiposity than the host strain, however it had lower adiposity (see **Fig 1**), the opposite result than predicted which is reflected in the large p-value.

Next we considered how well these patterns of strain differences meet our expectations based on the original QTL. For each, we expected that one or both sexes from one or both of the reciprocal strain pairs would be different from the host strain in the appropriate allelic direction at the corrected threshold (see **Table 1** to see the expected direction of effect). The matches between QTLs detected in F_2_ and consomic mice are shown visually on **Fig 1**, where appropriate bars are annotated with symbols for QTLs detected in the F_2_. As an example of specific expectations, for *Bwq6* we expect that the consomic strain with a B6 derived version of chr 9 (129.B6-Chr9) would be heavier (on average) than the host (129) strain and/or that the reciprocal strain (B6.129-Chr9) would be lighter than the host (B6) strain. Using this example, this prediction was correct, i.e., mice of the 129.B6-Chr9 strain weighed on average significantly more than did mice from 129 host strain; conversely, mice from the B6.129-Chr9 strain were lighter on average than were mice from the B6 host strain. In this example, both reciprocal strains and sexes differed in the expected direction, although we only required one of the two reciprocal strains or only one of the two sexes to differ to consider the QTL confirmed. Applying these criteria to the other statistical results in **Table 3**, each of the 8 QTL was confirmed (**Table 4**).

**Table 4:** QTL confirmation from the consomic strains. Confirmation criterion–one or more sexes and one or more reciprocal strains differed in the expected direction using a p-value threshold adjusted for multiple testing (see **Table 3**). *Bdln4* and *Bdln5* are combined because they are both on chromosome 9.

Count	QTL	Confirmed?
1	*Bwq5*	Yes
2	*Bwq6*	Yes
3	*Bdln3*	Yes
4,5	*Bdln4*,*5*	Yes
6	*Adip26*	Yes
7	*Adip27*	Yes
8	*Adip20*	Yes

We also evaluated whether sex-specific effects were consistent from the F_2_ to the consomic transition for these QTLs, although this evaluation did not impact the QTL confirmation process described above. Here the results were mixed. For *Bdln4* and *Bdln5*, the original QTLs studied in the F_2_ population were male-specific, with the B6 allele increasing body length (both of these body length QTLs are on chromosome 9 so the results for the consomic strain apply to both QTLs). True to expectations there was a difference between consomic and donor strain in body length for males (significant for B6.129-Chr9, and borderline for 129.B6-Chr9) but not in females (See **Table 3** for test statistics and **Table 5** for a summary of these sex-specific results). However, the sex-specific results from *Adip27* were not consistent with expectations. These expectations come from two studies of F_2_ populations that identify this QTL, and in both cases, the B6 allele in males but not females was associated with increased adiposity (see **Table 1** for references). However, here not only both male reciprocal consomic strains but also both female reciprocal consomic strains differed from the host either at a nominal (B6.129-Chr7 females) or adjusted threshold (129.B6-Chr7) in expected direction. Thus, the preservation of sex-specific QTLs from F_2_ to consomic was mixed, two were consistent and one was not. The detection of *Adip27* effect in consomic females but not F2 females is probably due to higher statistical power in consomics.

**Table 5:** Comparison of sex-specific QTLs between F_2_ crosses and consomic strains. QTL type was determined in F_2_ crosses.

QTL	Type	Consomics	Sex	Result	Consistent
*Bdln4*,*5*	Male-specific	129.B6-Chr9	F	x	Yes
			M	√	Yes
		B6.129-Chr9	F	x	Yes
			M	√√	Yes
*Adip27*	Male-specific	129.B6-Chr7	F	√√	No
			M	√√	Yes
		B6.129-Chr7	F	√	No
			M	√√	Yes

Differences between consomic and inbred host strains are indicated by:

x, p≥0.05 (no significant differences)

√, p<0.05 (a nominal p-value)

√√. p = 0.0545 (an adjusted p-value; **Table 3**).

In addition to verifying QTLs in these consomic strains, we also examined a consomic strain (donor chr 1) which had a substituted chromosome for which no body composition QTLs were reported from prior mapping studies. Thus we did not expect any difference in average body weight or length in the consomic strain compared to the host strain and the data confirmed this expectation (**Table 3**). This expectation of no-effect was the same one we had for adiposity, but here the expectation was not met. Instead, there *was* a female-specific QTL, with female consomic mice (129.B6-Chr1) being leaner than the females of the host strain.

Similarly, there were two new QTLs on chromosome 7, for body weight and length. In the original F_2_ studies, there was only an adiposity QTL (*Adip27*) on chromosome 7 but not a body weight or length QTL. But the host and consomic strains did differ in body weight and length (significant differences for both traits in B6.129-Chr7 males and for body length in 129.B6-Chr7 females).

There was another unexpected result. While the *Adip26* QTL was confirmed according to the criteria specified above (i.e., B6.129-Chr2 females were significantly fatter than B6 females), B6.129-Chr2 males differed from B6 males in the opposite direction (they were leaner). This deviation from expectation is marked in Fig 1 by the “&” symbol and possible explanations for this result are discussed below.

## Discussion

There are several methods to pursue QTL identification once they have been identified in intercrossed populations, and the use of congenic strains is a common one, e.g., [28]. However, this method is risky. QTLs might not always replicate in congenic strains but whether this might happen cannot be accurately predicted, in part because many potentially informative failures of QTL replication go unreported. These failures are especially troublesome because of the time and the costs involved in the production of congenic mice. Therefore, as a prelude to congenic construction and as way to manage risk, we tested whether the QTL was detectable in the appropriate consomic (whole chromosome substitution) strains. This is a cautious intermediate step for two reasons: first because the whole chromosome is the largest possible interval to capture the QTL and second, it allows us to tell whether the QTL can be detected on different type of genetic background. To be even more cautious, we bred reciprocals of each consomic strain, the logic being that some QTL might be more apparent on one host background relative to another.

To decide whether a QTL was confirmed, we had to establish criteria we could use to make this decision. We required the following two conditions be met: (i) there was a significant difference between mice from the consomic and inbred partner strains in one or more sexes and in at least one reciprocal strain, and (ii) the same direction of allelic effect in the consomic strain and in the original intercross population. (We reasoned that QTL might not be equally detectible in both reciprocal strains because of ceiling or floor effects). Overall we found that all eight original QTLs were detected in at least one sex or reciprocal strain, and several were detectable in both sexes and reciprocal strains (e.g., *Bwq6*). These eight QTLs would be logical targets of future positional cloning studies using the congenic approach, and it would also be logical to pick the host-donor combination that yielded the largest mean differences between strains in the hopes that these differences would be make certain aspects of congenic breeding easier.

There were two cases where the results were not what we predicted. *Adip26* was confirmed, but one of the strain groups differed markedly from expectations. (Males of the B6.129-Chr2 strain were supposed to be fatter than the host strain yet instead they were leaner). The second case was for *Adip27* –we expected there would be no difference in female mice between host and consomic groups yet there was. Several factors could explain these unexpected results. The polygenic nature of body composition increases the chance that more than one QTL (with opposing effects) maps to the same chromosome. For example, mouse Chr2 has many previously reported QTLs for adiposity including *Adip26* [29–35]. Epistatic effects are a potent force shaping obesity and related traits [19, 36, 37], and they can vary depending on genetic background and may interact with sex. Maternal (and more generally, parent-of-origin) effects also influence body size and composition QTLs [38, 39]. Finally, environmental effects could also have contributed to these unexpected results. Although mice used in the initial [19–21] and in the current study lived in the same facility, seemingly subtle environmental shifts over this time can have large effects on body composition [40, 41].

New QTLs were detected in the consomic strains that were not detected in the original intercross (on Chr 1 for adiposity and on Chr 7 for body weight and length). It may be that these QTLs were present in the earlier F_2_ studies but missed the statistical thresholds and thus were not reported. Alternatively, it could be that the homozygous genetic background of the consomic strains allow for more sensitive detection of some types of QTLs, including these. The increased sensitivity of consomic strains to detect body composition QTLs has been noted previously [42].

The main finding of this study was that eight of eight body size and composition QTLs originally detected in the intercrosses between B6 and 129 strains were confirmed in the consomic strains. This result agrees with a previously reported 88% of body composition QTLs replicated in new F_2_ populations of mice using the same breeding strategies and parental strains [43]. We and others have demonstrated that consomics strains are resources for the study of obesity [12, 45, 46]. The specific knowledge of which QTLs are detected in which reciprocal consomic strain and in which sex enables efficient planning of congenic mapping of these QTLs, which will minimize chances of failure of QTL retention and thus will facilitate their positional cloning. With increased emphasis on rigor and reproducibility of experimental outcomes in biomedical and behavioral research [38, 44], these results lay the foundation for congenic construction. Availability of this resource will enhance positional cloning not only in our laboratory, but may assist groups that use these or related strains in their QTL mapping studies, e.g., [47–50].

## Supporting Information

S1 Table List of markers used to genotype donor chromosome during construction of consomic strains. The chromosome genotyped (Chr), SNP marker name by rs number, and location in bp based on GRCm38.
(XLSX)

S2 Table Summary of genotyping of completed homozygous consomic strains for residual heterozygosity. The symbol of the strain tested and its catalog number (Mouse Mutant Regional Resource Center), number of markers tested, number of mice tested and the observed percent host genome in the non-target chromosomes.
(XLSX)
